# Use of In-Game Rewards to Motivate Daily Self-Report Compliance: Randomized Controlled Trial

**DOI:** 10.2196/11683

**Published:** 2019-01-03

**Authors:** Sara Taylor, Craig Ferguson, Fengjiao Peng, Magdalena Schoeneich, Rosalind W Picard

**Affiliations:** 1 Affective Computing Group Department of Media Arts and Sciences Massachusetts Institute of Technology Cambridge, MA United States; 2 Digital Accelerator Takeda Pharmaceuticals Cambridge, MA United States

**Keywords:** self-reports, protocol compliance, recreational games

## Abstract

**Background:**

Encouraging individuals to report daily information such as unpleasant disease symptoms, daily activities and behaviors, or aspects of their physical and emotional state is difficult but necessary for many studies and clinical trials that rely on patient-reported data as primary outcomes. Use of paper diaries is the traditional method of completing daily diaries, but digital surveys are becoming the new standard because of their increased compliance; however, they still fall short of desired compliance levels.

**Objective:**

Mobile games using in-game rewards offer the opportunity to increase compliance above the rates of digital diaries and paper diaries. We conducted a 5-week randomized control trial to compare the completion rates of a daily diary across 3 conditions: a paper-based participant-reported outcome diary (Paper PRO), an electronic-based participant-reported outcome diary (ePRO), and a novel ePRO diary with in-game rewards (Game-Motivated ePRO).

**Methods:**

We developed a novel mobile game that is a combination of the idle and pet collection genres to reward individuals who complete a daily diary with an in-game reward. Overall, 197 individuals aged 6 to 24 years (male: 100 and female: 97) were enrolled in a 5-week study after being randomized into 1 of the 3 methods of daily diary completion. Moreover, 157 participants (male: 84 and female: 69) completed at least one diary and were subsequently included in analysis of compliance rates.

**Results:**

We observed a significant difference (*F*_2,124_=6.341; *P*=.002) in compliance to filling out daily diaries, with the Game-Motivated ePRO group having the highest compliance (mean completion 86.4%, SD 19.6%), followed by the ePRO group (mean completion 77.7%, SD 24.1%), and finally, the Paper PRO group (mean completion 70.6%, SD 23.4%). The Game-Motivated ePRO (*P*=.002) significantly improved compliance rates above the Paper PRO. In addition, the Game-Motivated ePRO resulted in higher compliance rates than the rates of ePRO alone (*P*=.09). Equally important, even though we observed significant differences in completion of daily diaries between groups, we did not observe any statistically significant differences in association between the responses to a daily mood question and study group, the average diary completion time (*P=*.52), or the System Usability Scale score (*P*=.88).

**Conclusions:**

The Game-Motivated ePRO system encouraged individuals to complete the daily diaries above the compliance rates of the Paper PRO and ePRO without altering the participants’ responses.

**Trial Registration:**

ClinicalTrials.gov NCT03738254; http://clinicaltrials.gov/ct2/show/NCT03738254 (Archived by WebCite at http://www.webcitation.org/74T1p8u52)

## Introduction

Clinicians rely on self-reports to collect a variety of patient data (including mood or pain reports and descriptions of changing symptoms) for routine practice and during clinical trials. In many cases, patients are asked to complete paper diaries at regularly spaced prespecified times. However, it has been shown that patients often do not fill out diaries at the required time but instead fake compliance by filling them out later in batches [1]. Specifically, Stone et al found that only 11% of paper diaries were completed during the appropriate 30 min time window even though patients filled in 90% of the diaries [1].

Thus, it is critical to find a way to increase diary compliance. A common way to improve diary compliance is to transition from paper diaries to digital diaries. Even before smartphones were available, researchers were using palmtop computers to compare adherence rates between digital and paper diaries. Jamison et al had 36 participants monitor their pain daily for 1 year. They found that those participants that used digital means to record their pain completed an average of 71.5% (261/365) days, whereas those in the paper group only recorded their pain an average of 17.8% (65/365) days [2]. Palermo et al conducted a similar trial having 60 children report their pain over 7 days. They found that the digital group filled out significantly more diaries (average of 6.6/7 days or 94% completion) than the paper diary group (average of 3.8/7 days or 54% completion) [3].

With the rise of the smartphone and its near ubiquity (77% of Americans are now smartphone owners [4]), one of the simplest ways to increase diary compliance is to transition from paper diaries to digital diaries. These electronic diaries have compliance rates typically ranging from 60 to 80% over 4 weeks or less [5,6]. Experience sampling, also called ecological momentary assessment, is one of the methods used to gather an individual’s experiences in real time by asking them to stop what they are doing and record their experiences [7-9]. Nevertheless, this method is highly interruptive and cannot be used for long periods without losing participant engagement unless they are highly compensated.

Recently, researchers have started exploring the possibility of using game design techniques, especially mobile games, to increase compliance to various behaviors. Played on the ubiquitous smartphone, these games have captured the attention of a wide variety of demographics and are often targeted to specific subgroups to further increase game-playing compliance. In 2015, 51.3% of mobile phone users played a mobile game at least once per month, and this rate is expected to grow to 63.7% of mobile phone users by 2020 [10]. Furthermore, 77% of teens report playing mobile games on their mobile phone or tablet [11] and 85% of children who play mobile games play at least a few times a week [12]. Successful games use common design techniques and mechanics to produce a game loop that repeatedly draws players back on a regular schedule and encourages the player to watch ads, share on social media, or pay money to get special rewards in the game.

There are many definitions of what can be construed as a game; however, 1 common theme is that a game must provide a challenge or a goal that requires skill to overcome or achieve. Typically, games also provide rewards (often called in-game rewards) that have an inherent value to help the player overcome a challenge and achieve the end goal. On the other hand, there are countless examples of attempts at including game-like features, also known as gamification, into various programs and apps in an attempt to increase engagement with the system in question. Many gamification systems use points, badges, or leaderboards to try to encourage extended engagement. We note that gamification is distinct from a game as these points and badges do not directly help the player progress toward an in-game end goal; instead, they simply mark the individuals’ progress and provide no inherent value.

Some well-known examples of gamification include Fitbit [13], Apple Watch [14], Nike+ [15], Khan Academy [16], Wikipedia [17], Stack Overflow [18], Lyft [19], and many others. Although successful with some subgroups of people, these gamification systems all fail at engaging people for an extended period [20]. For example, Hanus et al included badges and leaderboards into an educational program and found that it led to lower satisfaction, lower motivation, and lower grades compared with the students who had the traditional educational program [21]. Anderson et al found that badges work when people are close to earning them, but then the activity of the user returns to baseline immediately after the badge is earned [22]. Other studies have shown that individuals are demotivated by leaderboards once they are behind [23,24]. In addition, Koivisto et al found that although everyone tires of simple gamification techniques, younger children tire faster than others, making the addition of true game design principles especially important in younger populations [25]. However, when a full game is developed and important game design principles such as fantasy, challenge, and curiosity [26] are implemented into a system, they become more engaging than their *gamification* counterparts.

Therefore, we hypothesize that using in-game rewards instead of gamification techniques will increase the diary completion compliance above simply transitioning to digital diaries. To test this hypothesis, we conducted a 5-week user study to compare the completion rates of a daily diary across 3 conditions: a paper diary, a digital diary, and digital diary with in-game rewards. Furthermore, as there are legitimate concerns that a game may influence how the participant answers the questions in the diary [27], we will also test the difference in the answer distributions between the different study groups.

## Methods

### Overview

In the following sections, we describe our novel game called “The Guardians” that was designed to increase compliance to a daily task. We also describe our experimental protocol and statistical methods.

### The Guardians: A Diary System With In-Game Rewards

**Figure 1:**
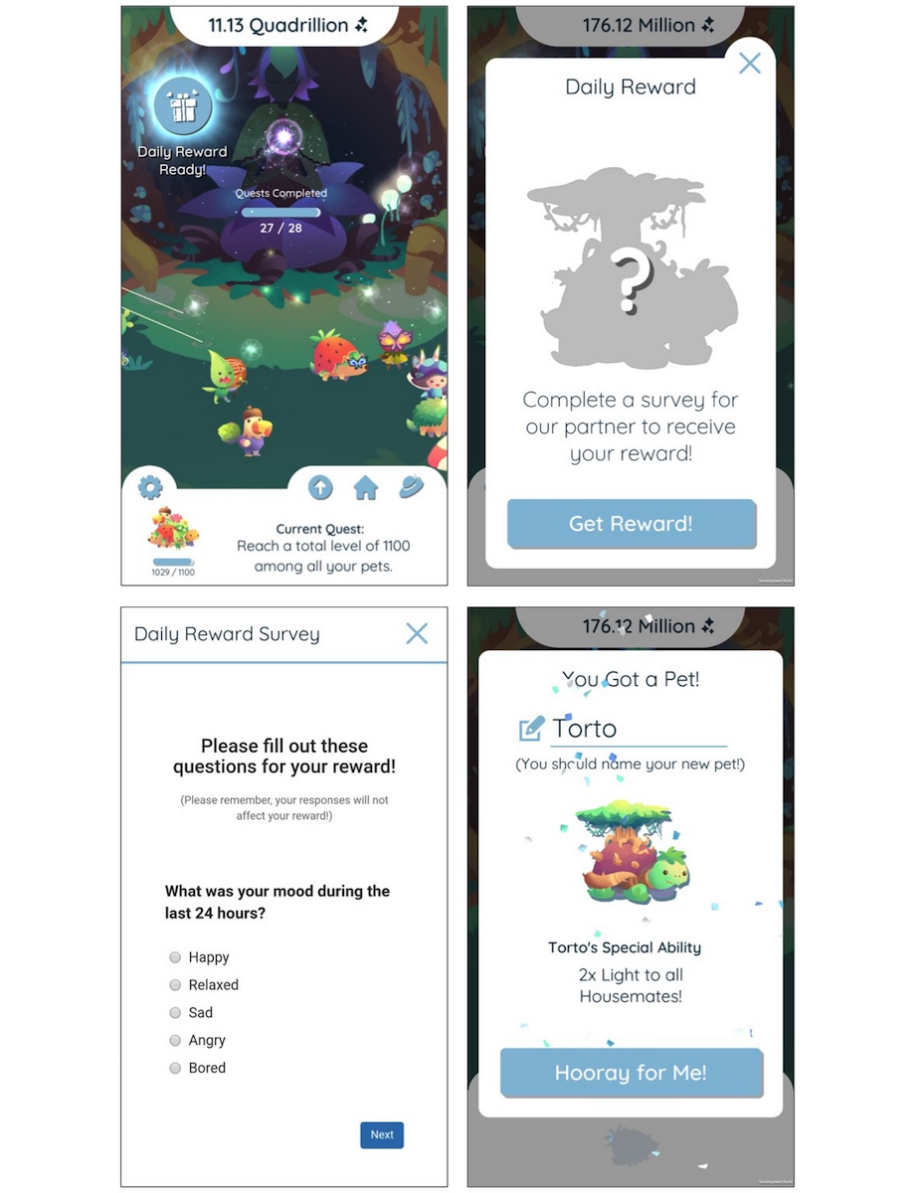
Screenshots of The Guardians while the player completes a daily diary. Players collect pets by filling out a daily diary. The pets are needed to complete quests and gather Light to awaken the guardian.

**Figure 2:**
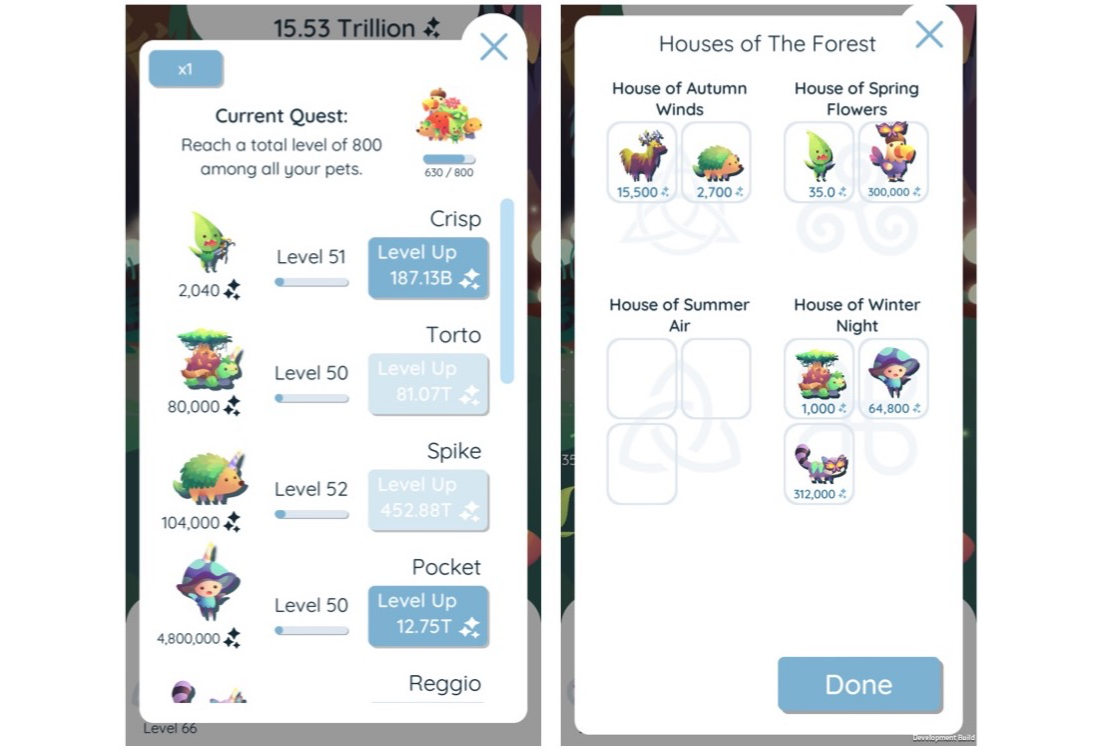
Screenshots of leveling menu and housing placement puzzle.

To test our hypothesis that in-game rewards can encourage engagement over a long period, we designed and developed a fully functional mobile game called “The Guardians” (see Figures 1 and 2) that was used to collect daily self-reported data.

To develop The Guardians, our team first conducted a series of pilot studies with potential users. We prototyped several different versions including several pen-and paper-based versions and simple text-based versions of the game to test the basic game mechanics. We conducted in-person interviews with children and adults (N=14; aged 7-50 years) and beta tested early prototypes to learn which game mechanics and art styles were most appealing. The final version of The Guardians used in this study was developed using Unity (Unity Technologies ApS), built for both the Android and iOS platforms, and incorporated the feedback learned from these early studies.

“The Guardians” is a mix of 2 popular game genres: idle games (a game that does not require constant player input to progress in the game but often progresses exponentially in response to a few user inputs [28], eg, Cookie Clicker and Adventure Capitalist) and pet collection games (a game where the player seeks to collect all of the pets in a set, eg, Pokemon or NekoAtsume). Besides being very popular, these genres are known for their ability to engage populations for long periods and for their appeal to a wide age range.

At the beginning of the game, the player is introduced to the Guardian, who asks for help in collecting Light to push back Darkness from the land. The player is given a pet and told that pets generate Light and is encouraged to care for them. Each day, the player has the opportunity to answer a set of diary questions and receive a reward, namely a new pet or an upgrade to an existing pet. Importantly, the player is reminded each time they start a diary that their diary responses do not affect their reward. This is reinforced by showing the silhouette of the reward before the player starts the diary, showing that it has not changed during the process (see Figure 1). This reminder is intended to encourage players to be honest in their answers instead of trying to get some response from the game. The only data returned by the diary to the game is a binary flag indicating that the diary has been completed.

The diary is accessed through the blue button in the top left of Figure 1. When the player is connected to the internet, they can click this button to open a diary. First, the silhouette of the reward is shown, and then a webview is opened showing the diary questions. When the player completes the diary, their answers are time-stamped and securely stored on an outside server, and the player is presented with their in-game reward. Importantly, the players do not have to fill out the diary to access the game nor do they have to play the game to access the diary. Thus, we have architected the system to try to avoid having the reward influence the content that people might enter into the diary while trying to simultaneously influence how often they go to fill out the diary.

After a diary entry is filled out and the player receives a pet as a reward, the pet helps the player generate Light for the Guardian, even when the app is closed. The player can customize, rename, and interact with the pets. Spending Light to level up the pets can increase the power of a pet. Players can also solve a simple placement puzzle each day for an additional bonus (see Figure 2 for screenshots of the leveling and placement puzzle menus). Along the way, the player completes several quests by reaching various levels for the pets. Once all of the quests are completed, the Guardian is awakened and Darkness is defeated. We designed “The Guardians” to be played in small periods of time (approximately 2-5 min each day) so that players would not tire quickly.

The version of the game used in this study was designed with enough content to engage the player for approximately 35 days, whereas the platform built to host the game is extensible for providing significantly longer experiments in the future and for enabling customized diary questions. The Guardians game was also designed to be accessible to all skill levels and to provide motivational content for a broad range of ages. Thus, 6-year-olds just beginning to read can enjoy receiving pets and interacting with them, whereas other optional aspects of the game, such as the positioning and leveling puzzles, are designed to be interesting and challenging for adults.

### Experimental Setup

Although Guardians was designed to increase compliance across a wide age range, the main focus of this paper is on individuals aged 6 to 24 years because this is the specific age group being targeted in an upcoming clinical trial. Before this new platform is used in a true clinical trial, we need to validate if the Game-Motivated electronic-based participant-reported outcome diary (ePRO) could improve daily adherence while not impacting the content of the responses. Thus, individuals aged 6 to 24 years were recruited via posters and internet postings to participate in a 5-week study about mood and mobile game habits in return for receiving a US $100 Amazon gift card at the end of the study. Recruitment was conducted from October 9, 2017, to November 15, 2017.

Interested individuals were screened for their age and ownership of a smartphone (only Android and iPhone owners were eligible for this study). Eligible individuals, and their parent/guardian if the individual was under 18 years, were emailed consent forms, which informed them of the study protocol, namely, that the study would consist of 3 parts:

1. A Web-based prestudy survey (approximately 30 min long) about demographics, general mood, and mobile game habits.
2. A diary on mood and mobile game habits (6 questions total) to be filled out daily for 5 weeks (35 days)—individuals were informed that they would be asked by study personnel to complete these diaries with an app or on paper.
3. A Web-based poststudy survey (approximately 20 min long) about general mood, the usability of the daily diary system, and qualitative feedback about the study.

Individuals were informed that they needed to complete the prestudy survey and at least four of the first seven diaries to be eligible for the first US $40 and that they could receive the remaining US $60 if they filled out the poststudy survey. No other requirements were made on filling out daily diaries beyond the minimum of 4 in the first week. Individuals who were asked to complete the daily diaries on paper were given an additional US $10 to cover the cost of mailing the diaries back to the study personnel.

Immediately after providing consent, but before completing the prestudy survey, participants were randomized without regard to age or gender into 1 of 3 groups for the daily diaries: a paper-based participant-reported outcome diary (Paper PRO), an ePRO, or our novel ePRO diary with in-game rewards (Game-Motivated ePRO). The 6 daily multiple-choice questions were identical for all 3 groups. Specifically, participants were asked to classify their mood, report on how much time they spent playing mobile games and how long they spent outside in the past 24 hours, how many ads they watched for in-game rewards, and what the weather (temperature and precipitation) was like in the past 24 hours (see Multimedia Appendix 1 for the exact survey content). Any diary that did not have all 6 questions completed was discarded. These questions were selected to study relationships between mood, mobile games, and the weather; however, this analysis is beyond the scope of this paper. Nevertheless, we note that it is common to ask individuals to answer short multiple-choice questions daily, and the mood question is of particular interest for health-related daily reports as it might be part of a clinical trial [29-31].

The paper PRO group also received 1 additional question. This question was used to confirm when the diary was completed and required viewing a Web page that displayed a word that changed every hour. Any paper diary with mismatched “word-of-the-hour” and recorded date were marked as incomplete. We note that 55% (23/42) participants in the Paper PRO group returned at least one diary with a mismatched “word-of-the-hour” and recorded date.

The ePRO group’s app was a simple native iOS or Android app that displayed a webview with the daily diary form. The Game-Motivated ePRO included the exact same webview and form. Therefore, the ePRO and Game-Motivated ePRO groups had identical daily diaries; however, the Game-Motivated ePRO participants received an in-game reward (ie, a pet) when they completed a daily diary, whereas the ePRO participants were just shown a screen thanking them for completing the diary and reminding them to return the next day. Participants in the Game-Motivated ePRO group were informed that they did not need to play the game to fill out their daily diary.

All participants were asked to complete the diary immediately after waking, but the diaries were available to complete from 3:30 am to 3:30 am each day (localized to the participant’s time zone). This time was chosen after learning that most college-aged individuals went to sleep between midnight and 3 am; thus, the diaries were reset while the majority of participants would be sleeping. Participants in the ePRO and Game-Motivated ePRO groups received a notification from their respective apps if they had not completed the diary by 9:30 am local time.

Participants were not informed that the main goal of the study was to measure daily diary compliance over the course of the study. Instead, they were told that the study was about mood, mobile game habits, and the weather because the daily questions referred to these topics. The entire study was done remotely and participants only had email contact with the study personnel.

The Massachusetts Institute of Technology (MIT) Committee on the Use of Humans as Experimental Subjects approved this protocol (MIT IRB Protocol #1708061907).

### Statistical Methods

#### Participants

We compared the individuals who were randomized but did not start the study and those who were randomized and did start the study (ie, filled out a prestudy survey) to see whether there were any age differences. As participants were blind to which study group they were in until after completing the prestudy survey, we hypothesized that there would be no significant differences found. Specifically, we conducted a 2-way analysis of variance (ANOVA) test to examine the interaction between choosing to participate and study arm on age. Tukey post hoc tests were conducted when statistically significant differences were found.

We hypothesized that there would be some portion of participants in each study arm that completed the prestudy survey, but did not complete any of the daily diaries. Specifically, we hypothesized that the Paper PRO group would have more participants that failed to complete a single daily diary than either the ePRO group or the Game-Motivated ePRO group as completing and returning diaries on paper presents a much larger barrier than completing diaries digitally. However, we did not expect a significant difference between the ePRO and Game-Motivated ePRO groups. To conduct this hypothesis test, we used the N-1 chi-square test in a pairwise manner to determine whether the study arms had a significantly different proportion of participants completing at least one daily diary. We adjusted for multiple comparisons by using the Bonferroni technique. We also computed the 95% CIs by using the adjusted Wald technique. Other than these tests, all of our other analyses, described below, focus on data from participants who completed at least one diary.

#### Game Engagement Analysis

To determine if participants in the Game-Motivated ePRO group actually engaged with the game during the study, we computed 4 measures of whether an individual engaged with the main components of the game (leveling pets, completing the placement puzzle, and interacting with the pets). Specifically, we measured (1) the average number of levels purchased daily, (2) the average number of daily housing placement changes, (3) the percentage of pets with a custom name, and (4) the average daily number of pets with an equipped cosmetic item.

To determine if higher levels of engagement correlated with the number of diaries completed, we analyzed the correlation between the number of diaries completed and the 4 engagement measures defined above. We also computed the correlation between age and these same engagement measures. In both correlation analyses, we adjusted for multiple comparisons by using the Bonferroni technique.

We are also interested in comparing the number of individuals engaged in the various components of the game; thus, we used the following thresholds to convert these measures into binary values. Individuals who were engaged with the leveling component of the game were expected to purchase at least 20 levels per pet per day as the exponential income players earn easily allows them to reach this amount. Furthermore, the minimum expected number of housing changes to optimally solve the housing placement puzzle was approximately 0.8 changes per day (some days required no changes). Therefore, we used an average of 0.5 housing placement changes per day (ie, one change every other day) as the engagement threshold for the housing puzzle. Finally, we used 50% as the engagement threshold for both the percentage of pets with a custom name and the average daily number of pets with a cosmetic item. Using these binary thresholds, we counted the number of participants who engaged with each component. We also computed the average diary completion for the combinations of engagement behaviors.

#### Daily Diary Completion

Beyond considering differences in the 3 study groups, we also examined age and gender differences on completion rates. Participants were split into 5 different age groups before any analyses were conducted. Specifically, the age groups were defined as children younger than 10 years—the average age of first ownership of a smartphone (ie, 6-9 years [32]), three 3-year age groups of preteens and teenagers (10-12 years, 13-15 years, and 16-18 years), and a group of young adults (19-24 years). Furthermore, some research has shown that there is a gender difference in how individuals engage with games [33,34]; therefore, we also examine gender effects in our analysis.

In some studies in which patient reported outcomes are collected, any data collected are useful regardless of which user it came from, but the more days of data collected the better. To determine the difference in average completion rates between the 3 study arms, we used a three-way ANOVA (also called a factorial ANOVA) test to consider how the diary method, age group, and gender influenced the adherence rate. Tukey post hoc tests are conducted when statistically significant differences are found.

On the other hand, some studies require high levels of completion to include an individual in the final analysis. If an individual falls below the required completion rate, study coordinators will often remove that participant from the study. Therefore, we compare the number of participants that are able to complete at least 90% of the daily diaries (ie, completing at least 32 of the 35 daily diaries). Specifically, we estimate the survival (ie, missing 3 or fewer diaries) of a participant in the study by computing the Kaplan-Meier curve [35] for each study group using the *survival* R package [36]. Then using the log-rank test [37], we can compare the survival curves of the 3 study groups. Specifically, it tests the hypothesis that at least 1 of the groups has a different survival than another against the null hypothesis that all of the groups have the same survival. If a significant *P* value is found, we will conduct a pairwise post hoc test to determine which pairs of study groups are significantly different from each other when adjusting for multiple comparisons using the Holm method [38]. Finally, we also compare the resulting number of participants with 90% or greater completion rates in each arm of the study using pairwise N-1 chi-square tests to check for significant differences in the participants that achieved 90% completion rate.

#### Self-Reported Response Distributions

As mentioned previously, the daily diaries contained the same 6 multiple-choice questions. A potential concern of the game is that it could affect the person’s state and cause them to respond differently to the questions [27]. We hypothesized that the method of completing the diary would not affect the answers to the mood, outside duration, number of ads watched, or the weather questions. Specifically, we hypothesized that the probability of selecting a particular answer would not differ between study arms after controlling for individual differences.

We used a Bayesian multinomial mixed effects logistic regression model in R, using the *brms* package [39], to estimate the association between the study arm and answer response. To account for clustering in the responses because of repeated measures for each individual, we included a random intercept grouped by the participant. We then computed point and interval (95% credibility) estimates of the predicted probability of selecting a particular answer response for each of the 3 study arms. If the credibility intervals of 2 study arms overlap for a given answer response, we conclude that no difference is detectable between the study arms. We complete this comparison for all pairs of study groups for each answer response. As this analysis is conducted in the Bayesian framework, we do not need to adjust for multiple comparisons.

We also performed the same analysis on the question about how long participants spent playing mobile games in the past 24 hours. We hypothesized that there would be a significant difference between the answers of the Game-Motivate ePRO group and the other groups because these individuals would be playing the Guardians regularly, especially in the 0 and 1 to 15 min responses as the game is designed to be played in approximately 5 min each day.

#### Average Duration of Daily Diary and System Usability Scale Scores

For the ePRO and Game-Motivated ePRO groups, we were able to track how long the participants took to complete each daily diary. Also, during the poststudy survey, participants in the ePRO and Game-Motivated ePRO evaluated the daily diary software using the System Usability Scale (SUS) [40]. As the diaries for these 2 groups were identical, we hypothesized that there would be no significant difference in the average duration or in the average SUS score between the 2 groups even when controlling for age and gender interactions using a three-way ANOVA test for each outcome measure. As before, Tukey post hoc tests were conducted when statistically significant differences were found.

#### Qualitative Poststudy Survey Results

Recall that at the end of the 35-day study, participants were given a Web-based poststudy survey to complete. Besides containing the SUS survey, participants were asked about what they thought the best and worst parts of the study were. Participants responded to these questions using an open format text response. These text responses were grouped into common themes and are presented in the results.

## Results

### Participants

**Figure 3:**
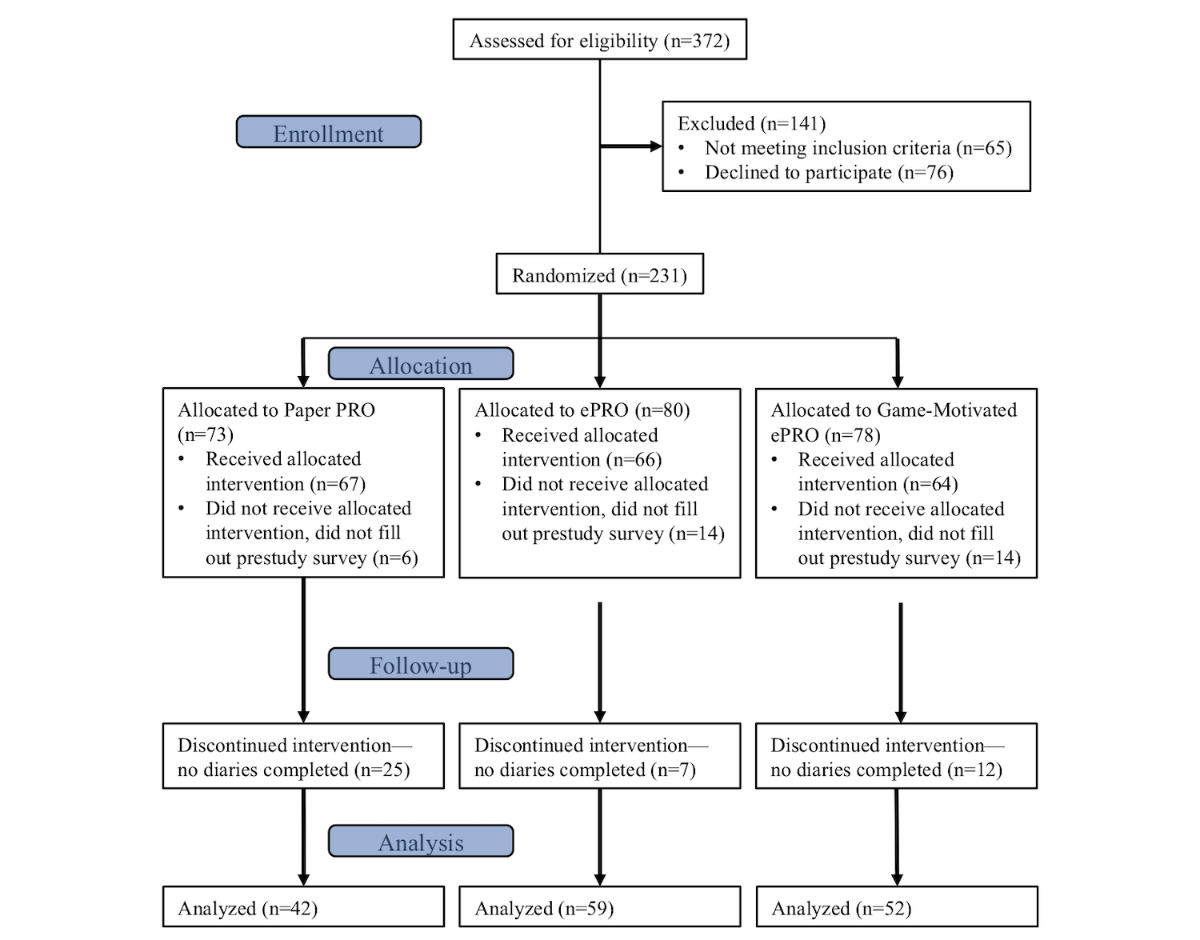
Flow of participants through the study. ePRO: electronic-based participant-reported outcome; PRO: participant-reported outcome.

**Figure 4:**
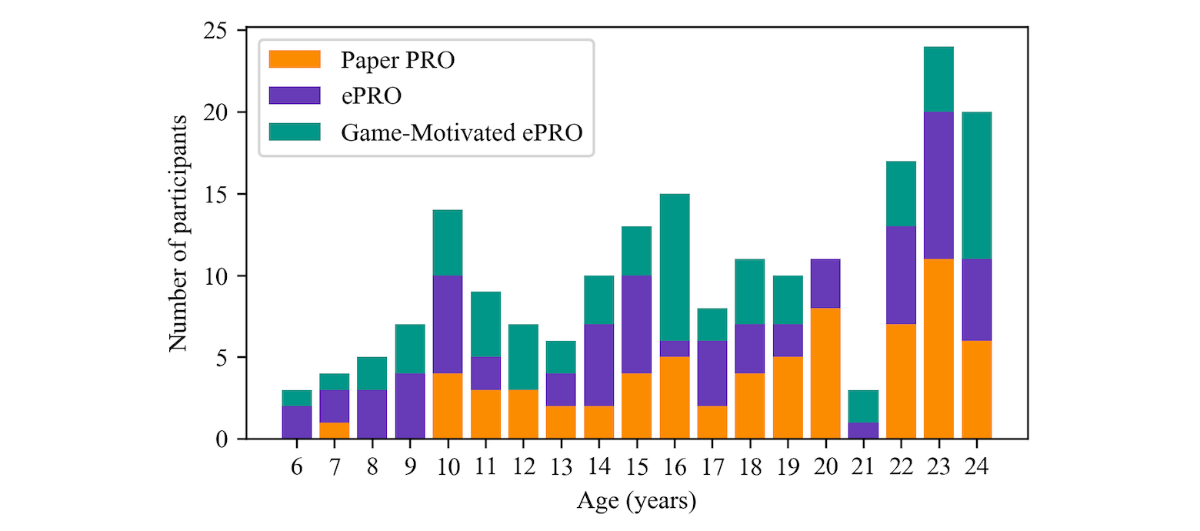
Distribution of participants who filled out the prestudy survey by age and daily diary condition. ePRO: electronic-based participant-reported outcome; PRO: participant-reported outcome.

Of the 372 individuals screened for the study, 309 individuals were eligible to participate and 232 individuals provided informed consent and were randomized into the 3 study groups. Of these, 197 completed the prestudy survey to begin the study: 67 participants (male: 31 and female: 36) in the Paper PRO group, 66 (male: 33 and female: 33) in the ePRO group, and 64 (male: 36 and female: 28) in the Game-Motivated ePRO group (see Figure 3 for the flow of participants in the study and Figure 4 and Table 1 for the distribution of study conditions in the different age groups).

The two-way ANOVA (Multimedia Appendix 1) did not reveal a statistically significant difference in age in the interaction between study group and participation in the study (*F*_2,226_=0.513; *P=*.60), as hypothesized. Nevertheless, we did observe a significant difference in age between study groups (*F*_2,226_=3.007; *P*=.05) and between individuals who completed the prestudy survey and those who did not (*F*_1,226_=3.184; *P*=.08). A Tukey post hoc test revealed that individuals randomized to the paper PRO group were slightly older (mean age difference 2.06 years; 95% CI 0.03-4.09 years; *P*_adj_=.05) and individuals who completed the prestudy survey were also slightly older (mean age difference 1.73 years; 95% CI −0.20 to 3.66 years; *P*_adj_=.08).

A total of 44 participants never completed a single daily diary after completing the prestudy survey. There was no significant difference (*P*_adj_=.57, 95% CI −20.2% to 32.9%) between the number of participants who never completed a diary in the ePRO group and Game-Motivated ePRO group; however, as hypothesized, there was a significant difference between the Paper PRO group and ePRO and Game-Motivated ePRO groups (*P*_adj_<.001; 95% CI 12.2%-39.7% and *P*_adj_=.06; 95% CI 3.0%-32.9%, respectively).

### Game Analysis

We found that the average number of levels purchased and the average number of pets with a cosmetic item were positively correlated with the number of diaries a participant completed (*P*_adj_<.001 and .02, respectively). The percentage of pets with custom names was found to be negatively correlated with the age of the participant (*P*_adj_=.09) and the average number of housing changes was found to be positively correlated with the age of the participant (*P*_adj_=.03, see Multimedia Appendix 1 for complete results).

**Table 1:** Demographic details of participants who completed at least one daily diary by study group.

Characteristic		PRO^a^ (n=42), n (%)	ePRO^b^ (n=59), n (%)	Game-Motivated ePRO (n=52), n (%)
**Gender**				
	Male	24 (57)	29 (49)	31 (60)
	Female	18 (43)	30 (51)	21 (40)
**Age (years)**				
	6 to 9	1 (2)	9 (15)	5 (10)
	10 to 12	10 (24)	7 (12)	8 (15)
	13 to 15	6 (14)	12 (20)	6 (12)
	16 to 18	5 (12)	7 (12)	14 (27)
	19 to 24	20 (48)	24 (41)	19 (37)

^a^PRO: participant-reported outcome.

^b^ePRO: electronic-based participant-reported outcome.

**Table 2:** Average diary completion per cluster of engagement (N=52).

Number of components engaged	n (%)	Daily diary completion, mean (SD)
All (n=4)	16 (31)	0.957 (0.051)
Three components	22 (42)	0.847 (0.198)
Two components	7 (14)	0.820 (0.141)
One component	5 (10)	0.657 (0.380)
None	2 (4)	0.971 (0.040)

**Figure 5:**
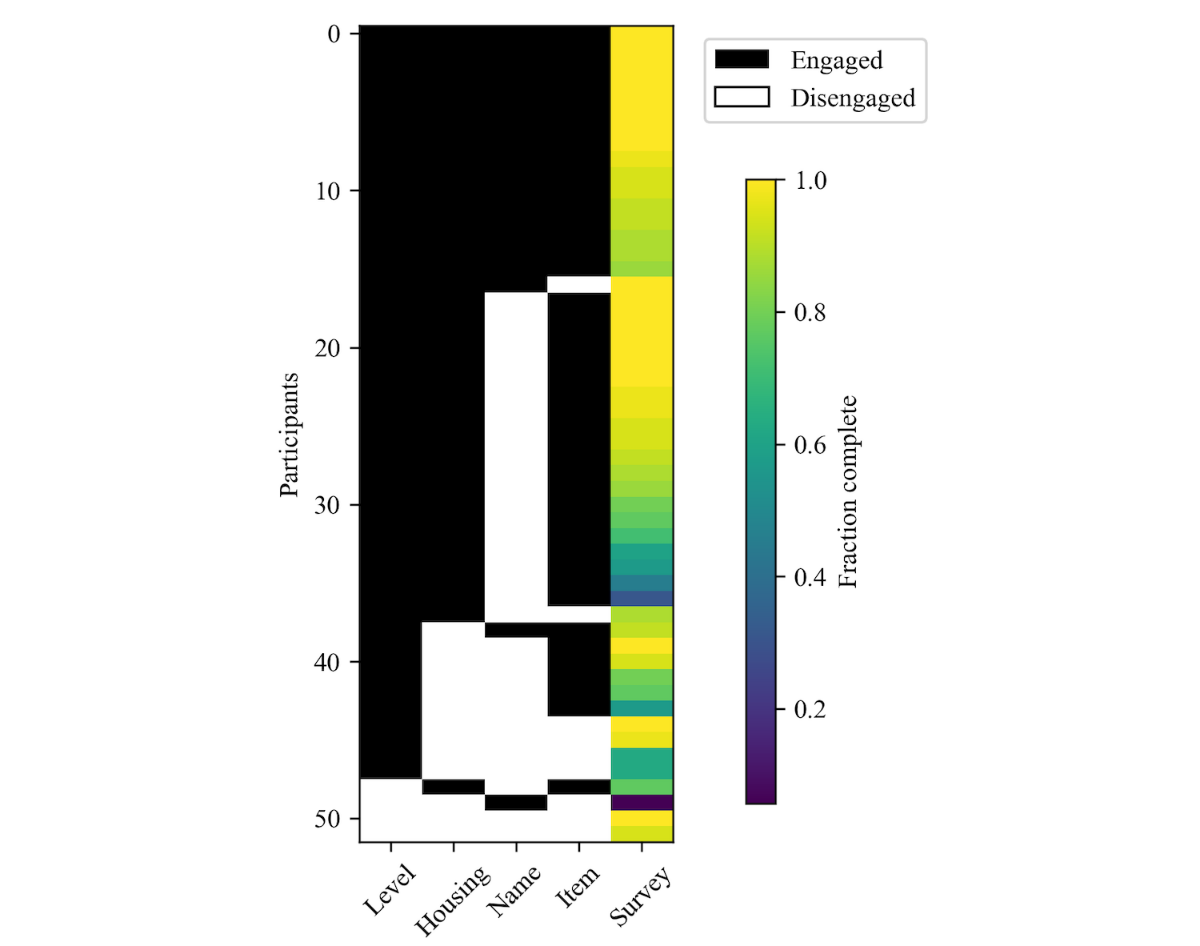
Game-motivated electronic participant-reported outcome participants’ engagement with various components of the game and the corresponding diary completion.

We found the majority of participants in the Game-Motivated ePRO group surpassed the binary thresholds to be considered *engaged* with the leveling component (48/52, 92%), equipping their pets with cosmetic items (43/52, 83%) and solving the housing placement puzzle (39/52, 75%). However, approximately one-third engaged in personalizing their pets with custom names (n=19, 37%). Looking at the combinations of engagement patterns (see Table 2 and Figure 5), we found that 16 participants engaged with all components of the game (16/52, 31%); these participants had very high daily diary completion rates (fraction completed: mean 0.957, SD 0.051). In addition, 2 of the participants did not engage with any components of the game; however, they still had very high daily diary completion rates (fraction completed: mean 0.971, SD 0.040).

### Daily Diary Completion

The Game-Motivated ePRO group had the highest compliance (mean completion 86.4%, SD 19.6%), followed by the ePRO group (mean completion 77.7%, SD 24.1%), and finally, the Paper PRO group (mean completion 70.6%, SD 23.4%).

**Figure 6:**
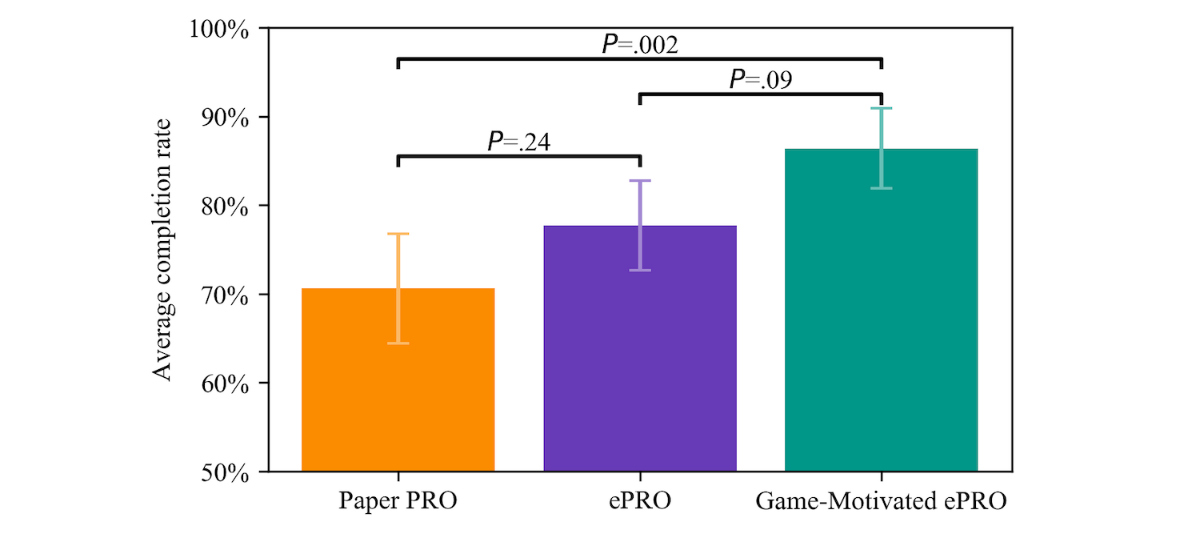
Average daily diary completion rate for each study arm. The error bars show the 95% CI and P values are from the Tukey posthoc test and are adjusted for multiple comparisons. ePRO: electronic-based participant-reported outcome; PRO: participant-reported outcome.

The three-way ANOVA (see Figure 6 and Multimedia Appendix 1) revealed a statistically significant difference in completion rates between the study groups (*F*_2,124_=6.341; *P*=.002) and a statistically significant interaction between study group and age group (*F*_8,124_=2.530; *P*=.01). A Tukey post hoc test revealed that the completion rate of the Game-Motivated ePRO was significantly higher than the Paper PRO (mean difference 15.8%; 95% CI 5.2%-26.4%; *P*_adj_=.002) and higher than the ePRO (mean difference 8.7%; 95% CI 1.0%-18.4%; *P*_adj_=.09). However, there was no statistically significant difference between the ePRO and Paper PRO groups (mean difference 7.1%; 95% CI −3.2% to 17.4%; *P*_adj_=.24). Therefore, our hypothesis that the Game-Motivated ePRO group would have significantly higher completion rates than both the ePRO group and the Paper PRO group was held. However, the hypothesis that the ePRO group would have a significantly higher completion rate than the Paper PRO did not hold.

**Figure 7:**
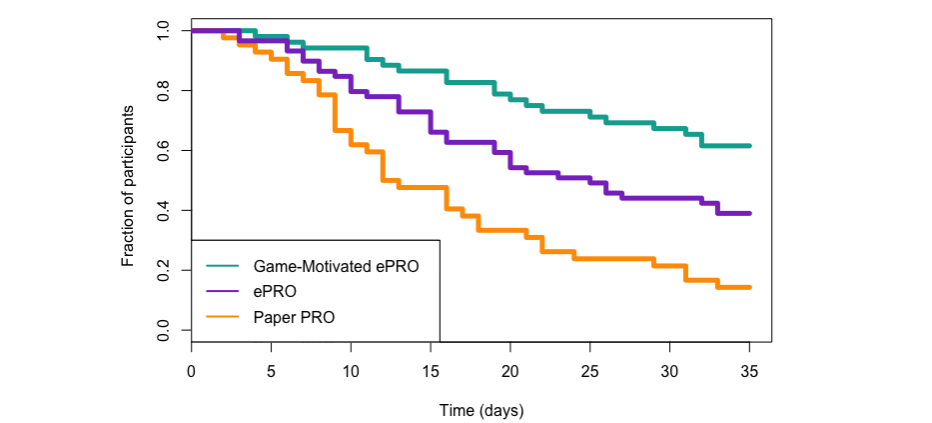
Kaplan-Meier curves of survival (ie, missing 3 or fewer diaries) for each study group. ePRO: electronic-based participant-reported outcome; PRO: participant-reported outcome.

The survival analysis estimated the Kaplan-Meier curves for each study group (see Figure 7) and found that the study groups were significantly different than each other (*P*<.001). A post hoc log rank test found that the Game-Motivated ePRO group had a significantly higher survival curve than the Paper PRO group (*P*_adj_<.001) and the ePRO group (*P*_adj_=.01). The ePRO group had a significantly higher survival curve than the Paper PRO group (*P*_adj_=.005).

**Figure 8:**
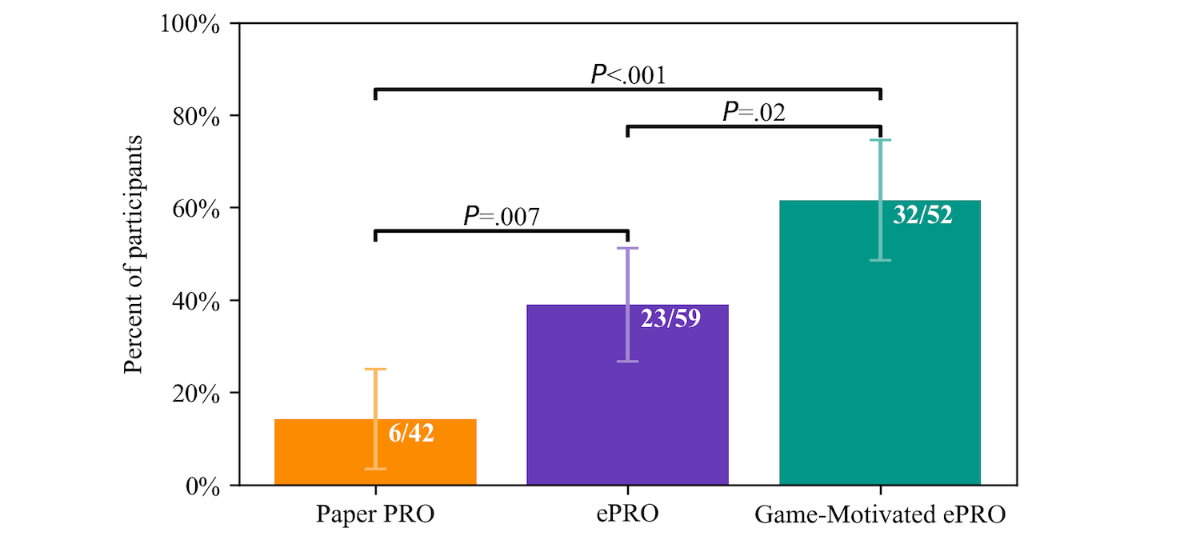
Percentage of participants who completed at least 90% of the daily diaries (ie, 32 out of 35 diaries) in each study arm. The 95% CI and P values are shown. ePRO: electronic-based participant-reported outcome; PRO: participant-reported outcomes.

Furthermore, as shown in Figure 8, there are significant differences in the final percentage of participants completing 90% of daily diaries between each of the study arms, with the Game-Motivated ePRO having the highest percentage, followed by the ePRO and Paper PRO, with all comparisons in the hypothesized directions having *P*<.02. We note that the increase in the percentage of participants with high compliance in the ePRO compared with the Paper PRO (CI 7.1%-39.8%) is almost identical to the increase in the percentage of participants with high compliance in the Game-Motivated ePRO compared with the ePRO (CI 3.9%-39.7%).

### Self-Reported Response Distributions

The results of the Bayesian multinomial mixed effects logistic regression models can be found in Multimedia Appendix 1. As all pairs of 95% credible intervals overlap for each answer response for the mood, number of ads watched, and weather questions, we conclude that no difference in the study arm has been detected for the answer selected. Therefore, there is no evidence that the probability of an answer response differs between the 3 study arms for these questions, as hypothesized. However, for the outside duration question, we note the Paper PRO and ePRO 95% credible intervals do not overlap for the “0 Minutes” response (Paper PRO: 3.3%-7.2% and ePRO: 7.3%-12.4%). Thus, we conclude that a statistically significant difference has been found in how these 2 study groups respond to this question of the daily survey.

Furthermore, we found a statistically significant difference between the ePRO and Game-Motivated ePRO groups in the probability of selecting “0 minutes” for the game duration question, as hypothesized. Specifically, we note that the 95% credibility interval for the probability of selecting “0 minutes” in the ePRO group (33.5%-49.7%) was significantly higher than the Game-Motivated ePRO group (13.0%-24.4%). We did not, however, see a significant difference in the “1-15 minute” response.

### Average Duration of Daily Diary

Our hypothesis that the average duration of time spent filling out the daily diary was not significantly different between the ePRO (mean duration 41.2 seconds, SD 19.3 seconds) and Game-Motivated ePRO (mean duration 44.1 seconds, SD 33.9 seconds) study groups was also confirmed (*F*_1,91_=0.423; *P*=.52). However, we did find some age and gender effects. The three-way ANOVA (see Multimedia Appendix 1) revealed a statistically significant difference in a participants’ average time spent filling out the daily diary between the age groups (*F*_4,91_=3.705; *P*=.008) and between the 2 genders (*F*_1,91_=4.256; *P=*.04). A Tukey post hoc test revealed that the 19- to 24-year-old participants’ average duration was significantly faster than the 16- to 18-year-old participants (mean difference 20.5 seconds; 95% CI 3.1-37.9 seconds; *P*_adj_=.01) and faster than the 10- to 12-year-old participants (mean difference 18.0 seconds; 95% CI −1.5 to 37.6 seconds; *P*_adj_=.09). Furthermore, male participants were found to be significantly faster than female participants (mean difference 9.1 seconds; 95% CI 0.2-18.0 seconds; *P*_adj_=.04).

### System Usability Scale Scores

We found that the usability of the diary, measured via SUS, was high for both the ePRO (mean 86.5, SD 14.5) and Game-Motivated ePRO (mean 86.9, SD 12.6). The three-way ANOVA (see Multimedia Appendix 1) revealed a significant difference between age groups (*F*_4,88_=2.221; *P*=.07) and between gender (*F*_1,88_=4.898; *P*=.03). A Tukey honestly significant difference test revealed that the only statistically significant (*P*_adj_<.1) difference in SUS score between age groups was between the participants aged 19 to 24 years and the 6- to 9-year-old participants, with the older participants rating the diary as more usable (mean difference 10.2; 95% CI −1.0 to 21.5; *P*_adj_=.09). In addition, we found that female participants rated the diary higher than male participants (mean difference 5.2; 95% CI 0.5-10.0; *P*_adj_=.03). Therefore, there was no significant difference in the SUS scores between the ePRO and Game-Motivated ePRO groups, as hypothesized.

### Qualitative Results From the Poststudy Survey

Over half of the participants (84/158) said the simplicity of the daily diaries and the opportunity it gave them to reflect on their day was the best part of the study. For example, 1 participant said “being able to reflect on the previous 24 hours” was the best part of the study. Another participant reported his or her favorite part was “each survey did not take long.” Moreover, 37 out of 56 (66%) of the Game-Motivated ePRO participants said the game was the best part. For example, one participant said their favorite part of the study was that “It was connected to a fun game, and it reminded me to take surveys.”

In total, 29.7% of participants (47/158) said remembering to do the daily diary was the worst part of the study. Nearly 40% of these participants (18/47) came from the Paper PRO group, with several participants suggesting that the diaries be converted to digital diaries that could be completed on a smartphone. For example, one participant suggested, “Instead of paper based this could have been mobile based.”

## Discussion

### Principal Findings

This study aimed to observe if there was a difference in daily diary completion in children and young adults between 3 different methods of diary, namely traditional paper-based diaries, digital diaries, and our custom game-motivated digital diary. As hypothesized, we observed significant differences in compliance to filling out daily diaries, with the Game-Motivated ePRO group having the highest compliance, followed by the ePRO group, and finally, the Paper PRO group. Nearly all individuals in the Game-Motivated ePRO group actually engaged with the various game mechanics, even though no such engagement was required to fill out the diaries. Importantly, there were no statistically significant differences in the content of the responses to 5 of the 6 daily questions, the average diary completion time, or in the SUS score between the ePRO and Game-Motivated ePRO groups. As mentioned previously, we expected the game duration question to have a significant difference in responses as the Game-Motivated ePRO group received a game to play during the study. Therefore, we conclude that the Game-Motivated ePRO method encouraged individuals to complete significantly more diaries without significantly altering the content of their responses.

Other researchers have also found promise in using in-game rewards to motivate individuals to complete a task. Cechanowicz et al added true game design techniques to the theme of a game show in their market research survey, including timers and points to add challenge and graphics to add to the game show theme. They found a significant difference in engagement between the full game and traditional survey and between the full game and the partial game (ie, when the challenge and graphical design elements were removed) [41]. Li et al created a full game to teach first time AutoCAD users how to use the software [42]. Participants who received the game-based version of the tutorial performed tests faster and reported higher subjective engagement levels than their traditional tutorial counterparts. Although their design is yet to be evaluated for engagement, Bindoff et al proposed a game design to help smokers regularly engage with smoking cessation content to earn currency in an idle world building game [43].

Some might worry that introducing a mobile game to increase compliance will introduce a potential *addiction* to the game that negatively influences the individual more than the benefit of reporting their symptoms. Whether a gaming addiction is officially recognized as a disorder or not, the game used in this study was designed to take only a few minutes to play and did not appear to have the negative effects one would expect from an addiction (eg, lack of control in playing the game, playing the game for excessive amounts of time, or being unable to stop playing even after negative consequences). For example, participants in the Game-Motivated ePRO group reported significantly lower rates of playing for 0 minutes a day than the other ePRO group but no differences for higher amounts of time. Thus, the game used in this study did not cause individuals to spend excessively more time playing games than the other groups. Nevertheless, future work in using games to motivate compliance should always monitor for the risks of potentially addictive behaviors.

### Limitations

Our study relied on having a significant compensation: US $40 to fill out 4 diaries in the first 7 days and an additional US $60 to complete a poststudy survey 35 days later. Ideally, using a daily diary system with in-game rewards would require little to no monetary compensation so that collecting data could be scaled to a massive number of participants without the need for large project budgets. Our “up to US $100” compensation might also account for the reason why the participants in the Paper PRO group had much higher completion rates than other studies (eg, mean completion rate of 70.6% in our study compared with 18% in the study by Jamison et al [2] and 54% in the study by Palermo et al [3]). Nevertheless, the compensation schedule in our study was designed to get individuals to start the habit of filling out the daily diaries and avoided offering compensation for each diary completed (a model used by many other daily diary studies).

As our study required individuals to own a smartphone, the proportion of the youngest participants was small in comparison with the proportion of young adults. Therefore, our results may not generalize well to individuals who rely on a parent or guardian owning a smartphone to complete an ePRO.

Although our study showed no difference in how the participants answer the questions in the daily diary, whether or not it was accompanied by the game, these results may not generalize to more complex diaries beyond the short, multiple-choice questions used in this study. Thus, future work should monitor how a game influences data collected in response to more complex diaries.

In addition, although we seek to capture long-term engagement in self-reporting compliance, this study was limited to a 5-week duration. Although this duration was longer than most daily diary studies, we have now extended our custom platform to provide content to engage individuals for longer periods of time in future studies, with the goal of understanding how engagement changes over months and through different periods of life.

Finally, although we posited that using a diary system as a part of a full game that provides in-game rewards will drive long-term engagement in a way that is not possible with simpler gamification techniques, a limitation of this study is that we do not know which elements of the game are, in fact, driving the better compliance. The novel full-narrative game has not been compared directly with other lesser gamification techniques. Future work should examine and deconstruct the various elements of the game to understand which elements contribute to increased motivation and compliance for daily diary reports.

### Conclusions

Self-reports are critical to research and clinical care; however, they require persistence and motivation to complete at regular intervals, especially if they involve answering daily diary questions about unexciting or possibly even unpleasant topics (such as those which are part of many clinical trials). Although moving away from traditional paper-based surveys to mobile digital surveys has shown increased compliance over traditional paper-based ones, and this finding was replicated here, our study shows that the compliance levels of today’s electronic diaries can still be improved.

We have shown that mobile game techniques, when properly implemented, can increase compliance in daily patient-reported outcomes. We have shown a significant increase in compliance over both a paper diary and a digital diary. We have shown data to support this hypothesis in a pediatric and young adult population. Furthermore, we have shown that the in-game rewards did not impact the content of the answers provided in the diaries.

Future work should seek to replicate these results when no monetary compensation is offered, in specific clinical patient populations to make sure that the difference in survey compliance rates holds, and for a period longer than 5 weeks. In addition, we intend to study the effects of engagement in older adult populations. Looking beyond patient reported outcomes and other surveys or self-reports, we also see potential for this motivation through games approach to be extended to other areas of health and well-being.

## Appendix

Multimedia Appendix 1 Supplementary material including daily survey questions and extended results.

Multimedia Appendix 2 CONSORT‐EHEALTH checklist (V 1.6.1).

## Abbreviations

ANOVA: analysis of variance
ePRO: electronic-based participant-reported outcome
MIT: Massachusetts Institute of Technology
PRO: participant-reported outcome
SUS: System Usability Scale

## References

[1] Stone AA, Shiffman S, Schwartz JE, Broderick JE, Hufford MR (2003). Patient compliance with paper and electronic diaries. Control Clin Trials.

[2] Jamison RN, Raymond SA, Levine JG, Slawsby EA, Nedeljkovic SS, Katz NP (2001). Electronic diaries for monitoring chronic pain: 1-year validation study. Pain.

[3] Palermo TM, Valenzuela D, Stork PP (2004). A randomized trial of electronic versus paper pain diaries in children: impact on compliance, accuracy, and acceptability. Pain.

[4] (2018). Pew Research Center.

[5] Harris C, Daniels K, Briner R (2003). A daily diary study of goals and affective well-being at work. J Occup Organ Psychol.

[6] Totterdell P, Kellett S, Teuchmann K, Briner R (1998). Evidence of mood linkage in work groups. Journal of Personality and Social Psychology.

[7] Csikszentmihalyi M, Larson R, Prescott S (1977). The ecology of adolescent activity and experience. J Youth Adolesc.

[8] Hernandez J, McDuff D, Infante C, Maes P, Quigley K, Picard R (2016). Wearable ESM: Differences in the experience sampling method across wearable devices. Proceedings of the 18th International Conference on Human-Computer Interaction with Mobile Devices and Services.

[9] Intille S, Haynes C, Maniar D, Ponnada A, Manjourides J (2016). μEMA: Microinteraction-based Ecological Momentary Assessment (EMA) Using a Smartwatch. Proceedings of the 2016 ACM International Joint Conference on Pervasive and Ubiquitous Computing.

[10] Statista.

[11] Statista.

[12] Statista.

[13] Kosecki D Fitbit.

[14] Apple.

[15] Nike.

[16] Khan Academy.

[17] Wikipedia.

[18] Stack Overflow.

[19] Lyft Blog.

[20] Hamari J, Koivisto J, Sarsa H (2014). Does Gamification Work? -- A Literature Review of Empirical Studies on Gamification. Proceedings of the 2014 47th Hawaii International Conference on System Sciences.

[21] Hanus MD, Fox J (2015). Assessing the effects of gamification in the classroom: a longitudinal study on intrinsic motivation, social comparison, satisfaction, effort, and academic performance. Comput Educ.

[22] Anderson A, Huttenlocher D, Kleinberg J, Leskovec J (2013). Steering user behavior with badges. Proceedings of the 22nd international conference on World Wide Web.

[23] Mejia J (2013). Grand Valley State University.

[24] Farzan R, DiMicco J, Millen D, Dugan C, Geyer W, Brownholtz E (2018). Results from deploying a participation incentive mechanism within the enterprise. Proceedings of the SIGCHI Conference on Human Factors in Computing Systems.

[25] Koivisto J, Hamari J (2014). Demographic differences in perceived benefits from gamification. Comput Human Behav.

[26] Malone T (1982). Heuristics for designing enjoyable user interfaces: Lessons from computer games. Proceedings of the 1982 Conference on Human Factors in Computing Systems.

[27] Lu AS, Baranowski J, Islam N, Baranowski T (2014). How to engage children in self-administered dietary assessment programmes. J Hum Nutr Diet.

[28] Alharthi S, Olaa A, Toups Z, Tanenbaum J, Hammer J (2018). Playing to Wait: A Taxonomy of Idle Games. Proceedings of the 2018 Conference on Human Factors in Computing Systems.

[29] Sano A, Taylor S, McHill AW, Phillips AJ, Barger LK, Klerman E, Picard R (2018). Identifying objective physiological markers and modifiable behaviors for self-reported stress and mental health status using wearable sensors and mobile phones: observational study. J Med Internet Res.

[30] Suhara Y, Yinzhan X, Pentland A (2017). DeepMood: Forecasting depressed mood based on self-reported histories via recurrent neural networks. Proceedings of the 26th International Conference on World Wide Web.

[31] Canzian L, Musolesi M (2015). Trajectories of depression: unobtrusive monitoring of depressive states by means of smartphone mobility traces analysis. Proceedings of the 2015 ACM International Joint Conference on Pervasive and Ubiquitous Computing.

[32] Influence Central.

[33] Ogletree SM, Drake R (2007). College students’ video game participation and perceptions: gender differences and implications. Sex Roles.

[34] Wright J, Huston A, Vandewater E, Bickham D, Scantlin R, Kotler J, Caplovitz A, Lee J, Hofferth S, Finkelstein J (2001). American children's use of electronic media in 1997: a national survey. J Appl Dev Psychol.

[35] Kaplan EL, Meier P (1958). Nonparametric estimation from incomplete observations. J Am Stat Assoc.

[36] Therneau T (2015). The Comprehensive R Archive Network.

[37] Mantel N (1966). Evaluation of survival data and two new rank order statistics arising in its consideration. Cancer Chemother Rep.

[38] Holm S (1979). A simple sequentially rejective multiple test procedure. Scand Stat Theory Appl.

[39] Bürkner P (2017). brms: an R package for Bayesian multilevel models using Stan. J Stat Soft.

[40] Brooke J, Jordan P, Thomas B, McClelland I, Weerdmeester B (1996). SUS-A quick and dirty usability scale. Usability Evaluation in Industry.

[41] Cechanowicz J, Gutwin C, Brownell B, Goodfellow L (2013). Effects of gamification on participation and data quality in a real-world market research domain. Proceedings of the First International Conference on Gameful Design, Research, and Applications.

[42] Li W, Grossman T, Fitzmaurice G (2012). GamiCAD: a gamified tutorial system for first time autocad users. Proceedings of the 25th annual ACM symposium on User interface software and technology.

[43] Bindoff I, de Salas K, Peterson G, Ling T, Lewis I, Wells L, Gee P, Ferguson SG (2016). Quittr: the design of a video game to support smoking cessation. JMIR Serious Games.

