# Supplementary material for: Use of In-Game Rewards to Motivate Daily Self-Report Compliance: Randomized Controlled Trial
Source: J Med Internet Res. 2019 Jan 3;21(1):e11683. doi: 10.2196/11683 (PMC6682282; doi:10.2196/11683)
Supplement: Multimedia Appendix 1 [file jmir_v21i1e11683_app1.pdf]

Supplemental Material

Use of In-game Rewards to Motivate Daily Self-Report Compliance

Sara Taylor, MS, Massachusetts Institute of Technology, Cambridge, MA, USA
Craig Ferguson, MS, Massachusetts Institute of Technology, Cambridge, MA, USA
Fengjiao Peng, MS, Massachusetts Institute of Technology, Cambridge, MA, USA
Magdalena Schoeneich, MBA, Takeda Pharmaceuticals, Cambridge, MA, USA
Rosalind W Picard, ScD, Massachusetts Institute of Technology, Cambridge, MA, USA

Correspondence: Sara Taylor (sataylor@media.mit.edu)
Phone: 612-860-8622
Massachusetts Institute of Technology
Media Lab, Affective Computing Group
77 Massachusetts Ave, E14-348V
Cambridge, MA 02142

Daily Diary Questions

Below is the daily diary administered to participants. All participants received questions 1-6 and were required to choose a single best response. Question 7 was only given to participants in the Paper PRO group.

Before running the 35-day study, we conducted informational interviews with people aged 6-50 years old to determine if any of the questions or responses should be modified. We note that originally only four moods were used based on choosing one representative word from each quadrant of the circumplex model of emotion that would be understandable for a wide age range, namely Happy, Relaxed, Sad, and Angry. The interview participants suggested that we include Bored as one of the moods. Thus, we included five moods total. No other questions were modified.

1) **What was your mood during the last 24 hours? (Choose one)**
   Happy Relaxed Sad Angry Bored

2) **How many minutes did you spend playing mobile games in the last 24 hours? (Choose one)**
   0 minutes 1-15minutes 16-30minutes 31-60 minutes 61+ minutes

3) **How many times did you watch ads for in-game rewards in the past 24 hours? (Choose one)**
   0 times 1 time 2-5 times 5-10 times more than 10 times

4) **How many minutes did you spend outside in the last 24 hours? (Choose one)**
   0 minutes 1-15minutes 16-30minutes 31-60 minutes 61+ minutes

5) **What has the weather been like in the last 24 hours? (Choose one)**
   Cold Just Right Hot

6) **What has the weather been like in the last 24 hours? (Choose one)**
   Sunny Cloudy Rainy Snowy

7) **What is displayed on the webpage (<URL Redacted>) right now?**

Participants Results

Table 1: 2-way ANOVA results to determine if average age is significantly different between study groups and completing the pre-study survey

	Df	SumSq	MeanSq	F-value	*P*-value
Study Group	2	171	85.47	3.007	**0.051**
Completed Pre-Study Survey	1	90	90.49	3.184	**0.076**
Study Group * Completed Pre-Study Survey	2	29	14.59	0.513	0.599
Residuals	226	6424	28.42		

Table 2: Tukey Post Hoc Tests for average age displaying only the comparisons with P-adj<0.1

		Mean Difference	Lower CI	Upper CI	*P-adj*
Study Group					
	Paper PRO - ePRO	2.06 years	0.03 years	4.09 years	0.046
Completed Pre-Study Survey					
	Completed – Did not complete	1.73 years	-0.20 years	3.66 years	0.078

Game Engagement Results

Table 3: Pearson correlations between game engagement and the total number of daily diaries completed and age of the participant

	Number of Daily Diaries Completed		Age	
	Pearson's ρ	*P*-value	Pearson's ρ	*P*-value
Average Levels Purchased	0.5894	**<0.001**	0.1009	0.477
Average Housing Changes	0.1992	0.157	0.2939	**0.034**
Percent Pets with Custom Name	0.0803	0.572	-0.2396	**0.087**
Average Number Pets with Item	0.3145	**0.023**	0.0494	0.728

Daily Diary Completion

Table 4: 3-way ANOVA results to determine if average completion rate is significantly different between study groups

	Df	SumSq	MeanSq	F-value	*P*-value
Study Group	2	0.587	0.29356	6.341	**0.002**
Age Group	4	0.166	0.04144	0.895	0.469
Gender	1	0.000	0.00010	0.002	0.964
Study Group * Age Group	8	0.937	0.11713	2.530	**0.014**
Study Group * Gender	2	0.034	0.01692	0.366	0.695
Age Group * Gender	4	0.209	0.05226	1.129	0.346
Study Group * Age Group * Gender	8	0.430	0.06137	1.326	0.244
Residuals	124	5.740	0.04629		

Table 5: Tukey Post Hoc Tests for average completion rate displaying only the comparisons with P-adj<0.1

		Mean Difference	Lower CI	Upper CI	*P-adj*

Study Group					
	Game-Motivated ePRO – Paper PRO	15.8%	5.2%	26.4%	0.002
	Game-Motivated ePRO – ePRO	8.7%	1.0%	18.4%	0.089
Study Group * Age Group					
	19-24 Game-Motivated ePRO - 19-24 Paper PRO	28.2%	4.3%	52.0%	0.007
	19-24 ePRO – 19-24 Paper PRO	21.4%	-1.1%	43.9%	0.083
	19-24 Game-Motivated ePRO – 6-9 ePRO	33.9%	3.8%	64.0%	0.013
	13-15 Game-Motivated ePRO – 6-9 ePRO	36.8%	-2.4%	76.0%	0.092
	19-24 ePRO – 6-9 ePRO	27.2%	-1.9%	56.2%	0.095

Self-Report Response Distributions

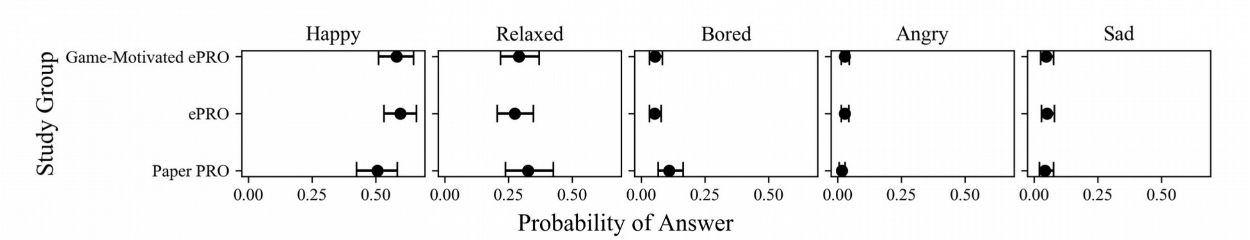

Figure 1: Probability and 95% credibility intervals of each response for the mood question by study group

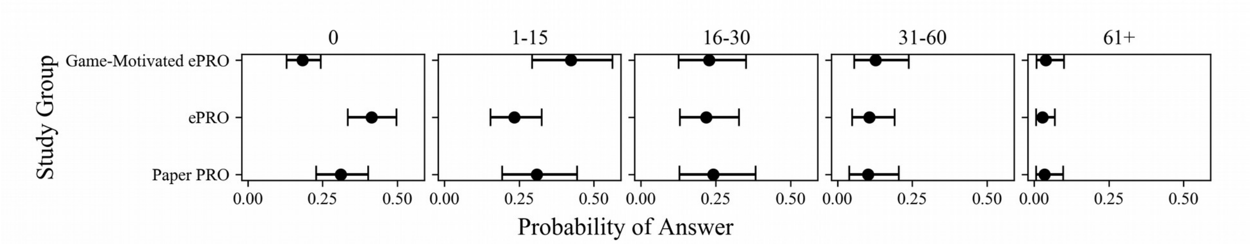

Figure 2: Probability and 95% credibility intervals of each response for the game duration question by study group

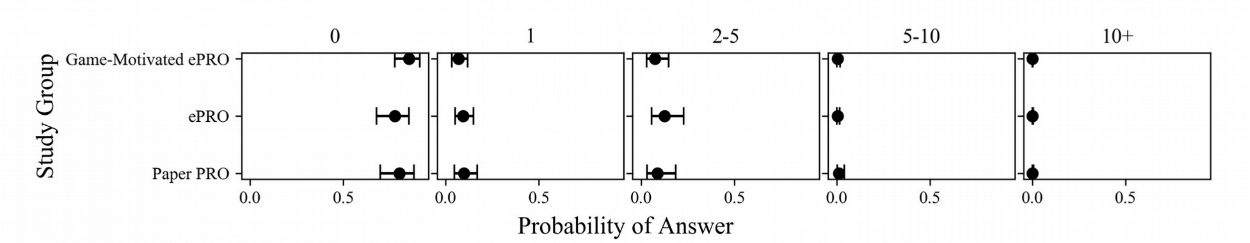

Figure 3: Probability and 95% credibility intervals of each response for the number of ads watched question by study group

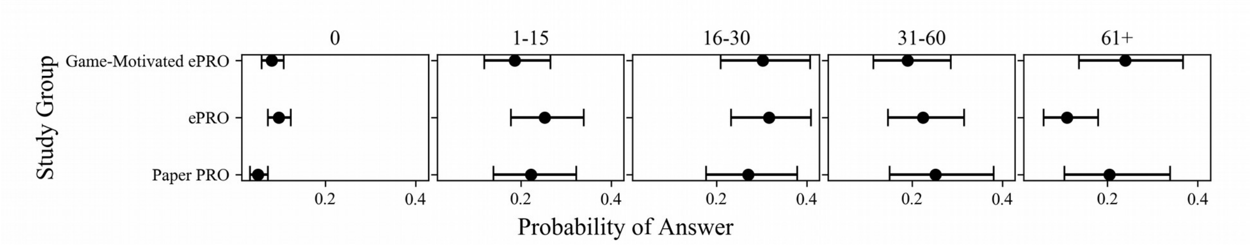

Figure 4: Probability and 95% credibility intervals of each response for the outside duration question by study group

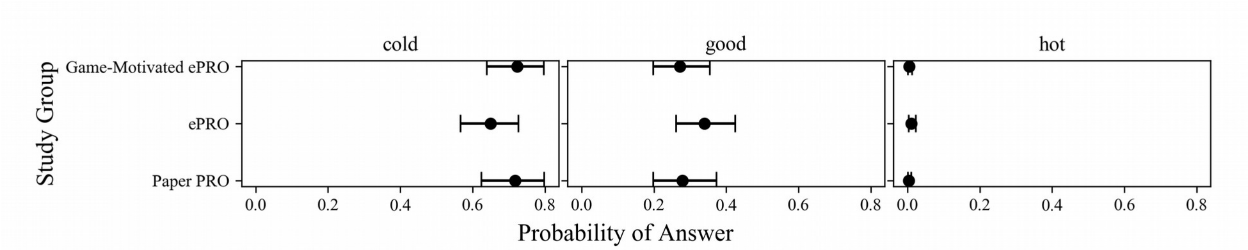

Figure 5: Probability and 95% credibility intervals of each response for the weather temperature question by study group

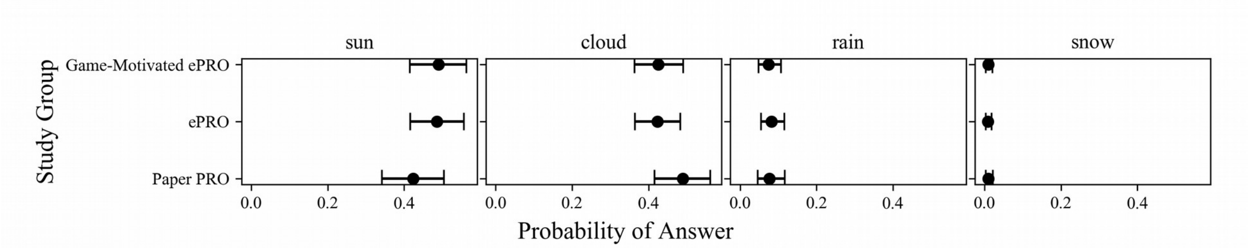

Figure 6: Probability and 95% credibility intervals of each response for the weather precipitation question by study group

Average Duration of Daily Diary

Table 6: 3-way ANOVA results to determine if average duration is significantly different between study groups

	**Df**	**SumSq, s**	**MeanSq, s**	**F-value**	***P*-value**
Study Group	1	233	232.7	0.423	0.517
Age Group	4	8153	2038.2	3.705	**0.008**
Gender	1	2341	2341.5	4.256	**0.042**
Study Group * Age Group	4	7583	1895.8	3.446	**0.011**
Study Group * Gender	1	2972	2971.8	5.402	**0.022**
Age Group * Gender	4	6160	1539.9	2.799	**0.031**
Study Group * Age Group * Gender	4	2881	720.3	1.309	0.273
Residuals	91	50065	550.2		

Table 7: Tukey Post Hoc Tests for average duration displaying only the comparisons with P-adj<0.1

		**Mean Diff, s**	**Lower CI, s**	**Upper CI, s**	***P-adj***
Age Group					
	19-24 years old – 16-18 years old	20.5	3.1	37.9	0.012
	19-24 years old – 10-12 years old	18.0	-1.5	37.6	0.086
Gender					
	Males – Females	9.1	0.2	18.0	0.044
Study Group * Age Group					
	19-24 ePRO – 16-18 Game-Motivated ePRO	30.3	4.7	55.9	0.008
	19-24 ePRO – 10-12 ePRO	37.4	4.7	70.1	0.012
	19-24 Game-Motivated ePRO – 16-18 Game-Motivated ePRO	27.4	0.6	54.2	0.040
	19-24 Game-Motivated ePRO – 10-12 ePRO	34.5	0.9	68.1	0.039
Study Group * Gender					
	Male Game-Motivated ePRO – Female Game-Motivated ePRO	20.4	-3.0	37.7	0.014
Age Group * Gender					

	Males 19-24 – Females 16-18	39.0	11.3	55.7	<0.001
	Females 19-24 – Females 16-18	35.9	7.0	64.7	0.004
	Males 16-18 – Females 16-18	36.0	2.7	69.3	0.023
	Males 13-15 – Females 16-18	32.9	-1.2	67.1	0.069

System Usability Scale Scores

Table 8: 3-way ANOVA results to determine if SUS score is significantly different between study groups

	**Df**	**SumSq**	**MeanSq**	**F-value**	***P*-value**
Study Group	1	4	3.7	0.024	0.877
Age Group	4	1370	342.5	2.221	**0.073**
Gender	1	755	755.3	4.898	**0.030**
Study Group * Age Group	4	1974	493.4	3.200	**0.017**
Study Group * Gender	1	617	616.7	3.999	**0.049**
Age Group * Gender	4	95	23.8	0.154	0.961
Study Group * Age Group * Gender	4	1403	350.8	2.275	**0.068**
Residuals	88	13570	154.2		

Table 9: Tukey Post Hoc Tests for SUS score displaying only the comparisons with P-adj<0.1

		**Mean Diff**	**Lower CI**	**Upper CI**	***P-adj***
Age Group					
	19-24 years old – 6-9 years old	10.2	-1.0	21.5	0.094
Gender					
	Female – Male	5.2	0.5	10.0	0.032
Study Group * Age Group					
	19-24 ePRO – 6-9 ePRO	20.5	3.1	37.8	0.008
	19-24 Game-Motivated ePRO – 6-9 ePRO	19.0	1.1	36.8	0.028
	6-9 Game-Motivated ePRO – 6-9 ePRO	22.9	0.7	46.5	0.065
Study Group * Gender					
	Female ePRO – Male ePRO	9.7	1.0	18.3	0.022
Study Group * Age Group * Gender					
	Female 19-24 ePRO – Male 6-9 ePRO	28.9	2.6	54.9	0.017
	Female 19-24 ePRO – Male 16-18 ePRO	45.0	-2.2	92.2	0.081
